# Supplementary material for: Transcriptional Remodeling Patterns in Murine Dendritic Cells Infected with Paracoccidioides brasiliensis: More Is Not Necessarily Better
Source: J Fungi (Basel). 2020 Nov 24;6(4):311. doi: 10.3390/jof6040311 (PMC7712260; doi:10.3390/jof6040311)
Supplement: Supplementary file 1 [file jof-06-00311-s001.zip › suplemmental material/Supplemental Figures.docx]

**Supplemental figures**


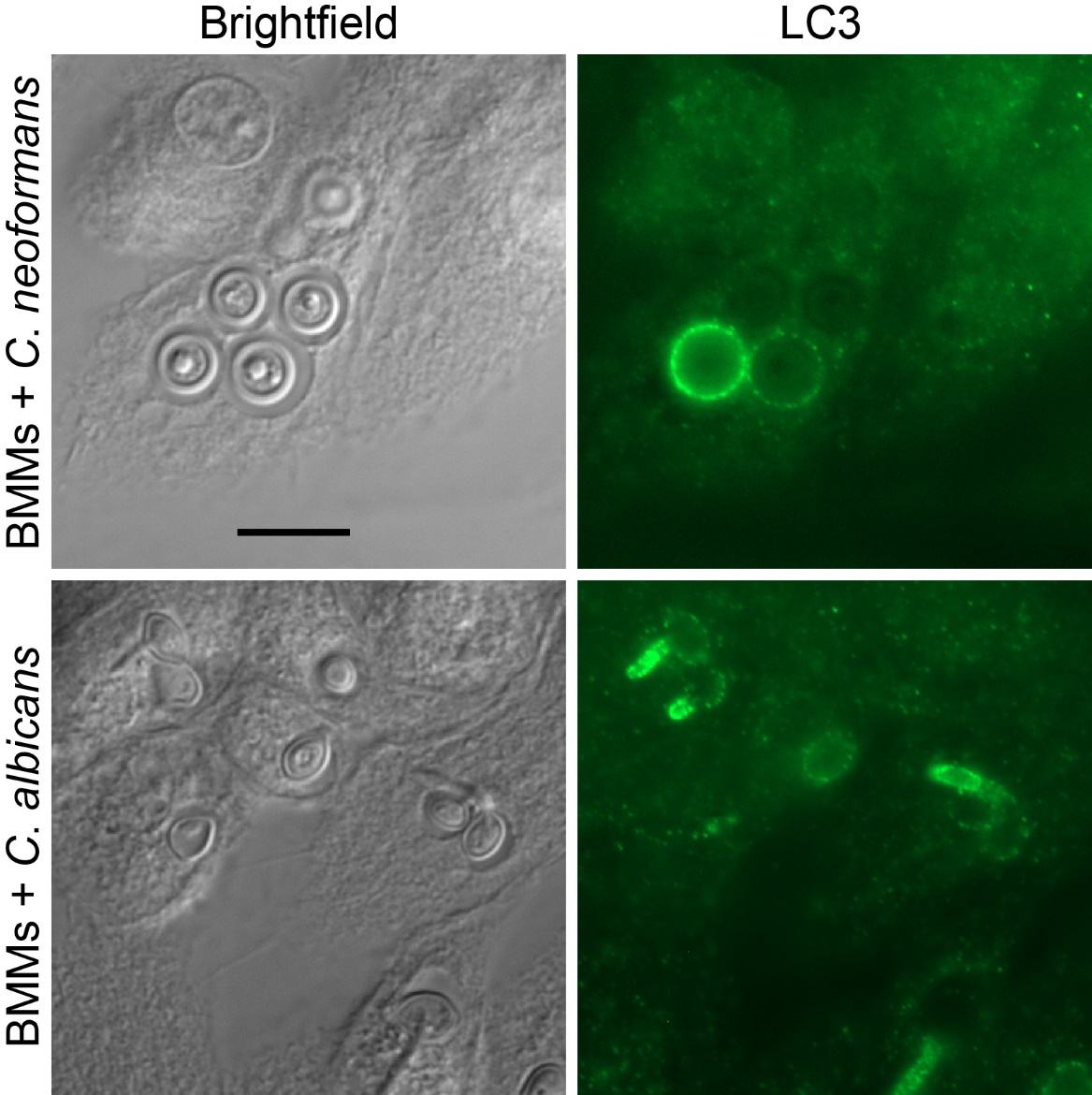


**Figure S1.** LAP in BMMs infected with *C. neoformans* and *C. albicans*. As positive controls for experiments shown in Figure 7, we performed a similar experiment with two fungi that had been previously detected as targets for macrophage LAP. The experiments were made with BMMs from the BALB/c mouse strain, which were co-incubated with fungi for 12 h. *C. neoformans* cells had been previously opsonized with the monoclonal IgG1 18B7.





**Figure S2.** **Transcripts levels detected by RNA-seq compared to RT-qPCR assay.** Murine bone-marrow derived DCs from A/J and B10.A mouse strains were infected by *P. brasiliensis* for 6 h and the same total RNA used in the RNA-seq was employed in RT-qPCR assays. The primers sequences are described in Table S1 and all assay conditions are as described in the Materials and Methods section**.** The analyzed genes encode cytokines (IL1β, IL6, IL10 and TNF-α), chemokines (CCL22 and CXCL10), the transcription factor NF-κB1 (p105) and the adapter molecule MyD88.





**Figure S3. Cytokines and chemokine profile from *P*. *brasiliensis*-infected Dendritic cells.** The supernatants of bone-marrow derived DCs from A/J and B10.A mouse strains after 6 h of infection with *P. brasiliensis* were quantified by ELISA assay, as described in the Materials and Methods section**.**
