# Supplementary material for: Transcriptional Remodeling Patterns in Murine Dendritic Cells Infected with Paracoccidioides brasiliensis: More Is Not Necessarily Better
Source: J Fungi (Basel). 2020 Nov 24;6(4):311. doi: 10.3390/jof6040311 (PMC7712260; doi:10.3390/jof6040311)
Supplement: Supplementary file 1 [file jof-06-00311-s001.zip › suplemmental material/Table S9. Comparison of the modulation of selected fungal immune-related genes.docx]

**Table S9. Comparison of the modulation of selected fungal immune-related genes in *P. brasiliensis* infected dendritic cells from A/J and B10.A mice strains.** The genes presented are based in the KEGG database and other sources.

| Gene | Name | Fold change* | |
| --- | --- | --- | --- |
|  |  | **A/J** | **B10.A** |
| Pattern of Recognition Receptor (PRR) |  |  |  |
| TLR8 | Toll-like receptor 8 | -1.24 | **-1.49** |
| TLR9 | Toll-like receptor 9 | 1.20 | **-1.90** |
| Clec2d | C-type lectin domain family 2, member d | 1.37 | **1.47** |
| Clec4e (Mincle) | C-type lectin domain family 4, member e | **2.36** | **3.63** |
| Clec4d | C-type lectin domain family 4, member d | 1.53 | **1.67** |
| Clec5a (MDL-1) | C-type lectin domain family 5, member a | 1.08 | **2.30** |
| Clec1b | C-type lectin domain family 1, member b | -1.04 | **-1.92** |
| Clec4a1 | C-type lectin domain family 4, member a1 | -1.08 | **-1.53** |
| Clec4a2 | C-type lectin domain family 4, member a2 | -1.10 | **-1.46** |
| Clec4a3 | C-type lectin domain family 4, member a2 | -1.11 | **-1.72** |
| Clec9a | C-type lectin domain family 9, member a | -1.22 | **-2.26** |
| Clec10a | C-type lectin domain family 10, member A | -1.12 | **-2.42** |
| Mrc1 (MR) | Mannose receptor, C type 1 | -1.19 | **-1.92** |
| Nlrp1b | NLR family, pyrin domain containing 1b | 1.13 | **-1.83** |
| Nlrp3 | NLR family, pyrin domain containing 3 | 1.18 | **1.91** |
| Nlrp10 | NLR family, pyrin domain containing 10 | -1.24 | **-1.78** |
| Scarf1 | Scavenfer receptor class F, member 1 | 1.17 | **1.97** |
| Scarb1 | Scavenger receptor class B, member 1 | -1.08 | **-1.48** |
| Itgb2l | Integrin beta 2-like | -1.12 | **1.43** |
| CD14 | CD14 antigen | 1.32 | **1.87** |
| Colec12 | Collectin sub-family member 12 | -1.10 | **-1.57** |
| PRR signal Transduction |  |  |  |
| Casp2 | Caspase 2 | -1.18 | **-1.63** |
| Casp4 | Caspase 4 | **1.83** | **2.27** |
| Casp6 | Caspase 6 | -1.02 | **-1.43** |
| Casp7 | Caspase 7 | 1.09 | **1.82** |
| Mapk8 | Mitogen-activated protein kinase 8 | 1.00 | **1.68** |
| Mapkapk2 | MAP kinase-activated protein kinase 2 | 1.35 | **1.72** |
| MyD88 | Myeloid differentiation primary response gene 88 | 1.16 | **1.42** |
| Bcl3 | B-cell leukemia/lymphoma 3 | **1.60** | 1.38 |
| Malt1 | Mucosa associated lymphoid tissue lymphoma translocation gene 1 | 1.26 | **2.76** |
| Traf1 | TNF Receptor Associated Factor 1 | **2.30** | **2.17** |
| Traf5 | TNF Receptor Associated Factor 5 | 1.12 | **1.85** |
| Traf6 | TNF Receptor Associated Factor 6 | 1.20 | **1.44** |
| CD40 | CD40 antigen | **2.57** | **5.29** |
| CD70 | CD70 antigen | **2.80** | **5.56** |
| CD80 | CD80 antigen | 1.35 | **2.24** |
| CD83 | CD83 antigen | 1.74 | **1.83** |
| CD86 | CD86 antigen | 1.21 | **1.50** |
| Transcription factor  and other proteins |  |  |  |
| Irf1 | Interferon regulatory factor 1 | **1.84** | **1.91** |
| Irf4 | Interferon regulatory factor 4 | 1.16 | **1.51** |
| Irf7 | Interferon regulatory factor 7 | **2.99** | **3.46** |
| Irf9 | Interferon regulatory factor 9 | **1.75** | **1.64** |
| Map2k1 (MKK1) | Mitogen-activated protein kinase kinase 1 | 1.16 | **1**.**48** |
| Nfkbia | Nuclear factor of kappa light polypeptide gene enhancer in B-cells inhibitor, alpha | 1.57 | **1.76** |
| Nfkb1 | Nuclear Factor Kappa B Subunit 1 | 1.36 | **1.74** |
| Nfkb2 | Nuclear Factor Kappa B Subunit 2 | **1.61** | **1.62** |
| Nfkbiz | Nuclear factor of kappa light polypeptide gene enhancer in B-cells inhibitor, zeta | **2.64** | 2.10 |
| Tfeb | Transcription factor EB | -1.01 | **-1.63** |
| *Cytokines* |  |  |  |
| Csf1 (M-CSF) | Colony stimulating factor 1 (macrophage) | 1.32 | **1.92** |
| Csf3 (G-CSF) | Colony stimulating factor 3 (granulocyte) | 4.32 | **9.52** |
| Il1a | Interleukin 1 alpha | 1.58 | **5.51** |
| Il1b | Interleukin 1 beta | **1.96** | **3.37** |
| Il6 | Interleukin 6 | **2.53** | **5.52** |
| Il10 | Interleukin 10 | **9.32** | **23.73** |
| Il12b (Il12p40) | Interleukin 12B | 6.95 | **6.05** |
| Il16 | Interleukin 16 | -1.24 | **-1.79** |
| Il23a | Interleukin 23, alpha subunit p19 | 4.84 | **3.77** |
| Il27 | Interleukin 27 | 1.61 | **3.66** |
| Tnf | Tumor necrosis factor alpha | **2.41** | **4.75** |
| *Chemokines* |  |  |  |
| Ccl1 | Chemokine (C-C motif) ligand 1 | **2.07** | - |
| Ccl3 | Chemokine (C-C motif) ligand 3 | 1.34 | **2.73** |
| Ccl4 | Chemokine (C-C motif) ligand 4 | 1.44 | **3.28** |
| Ccl5 | Chemokine (C-C motif) ligand 5 | **1.87** | **2.24** |
| Ccl9 | Chemokine (C-C motif) ligand 9 | 1.07 | **1.58** |
| Ccl12 | Chemokine (C-C motif) ligand 12 | 1.21 | **2.38** |
| Ccl17 | Chemokine (C-C motif) ligand 17 | 1.63 | **2.32** |
| Ccl22 | Chemokine (C-C motif) ligand 22 | **2.14** | **3.39** |
| Ccl24 | Chemokine (C-C motif) ligand 24 | **1.44** | **2.01** |
| Cxcl1 (KC) | Chemokine (C-X-C motif) ligand 1 | **4.22** | **6.04** |
| Cxcl2 | Chemokine (C-X-C motif) ligand 2 | **2.73** | **4.12** |
| Cxcl3 | Chemokine (C-X-C motif) ligand 3 | **3.47** | **8.24** |
| Cxcl5 | Chemokine (C-X-C motif) ligand 5 | - | **3.23** |
| Cxcl9 | Chemokine (C-X-C motif) ligand 9 | - | **3.12** |
| Cxcl10 (IP-10) | Chemokine (C-X-C motif) ligand 10 | 2.50 | **2.98** |
| Cxcl14 | Chemokine (C-X-C motif) ligand 14 | -1.09 | **1.69** |
| *Other proteins* |  |  |  |
| Ptgs2 (Cox-2) | Prostaglandin-endoperoxide synthase 2 | **2.48** | **10.79** |
| Ptx3 (TSG-14) | Pentraxin-related protein 3 | 1.17 | **2.75** |
| Stat1 | Signal transducer and activator of transcription 1 | **1.88** | **2.34** |
| Stat2 | Signal transducer and activator of transcription 2 | **1.66** | **1.95** |
| Stat4 | Signal transducer and activator of transcription 4 | **1.63** | **1.65** |
| Stat5a | Signal transducer and activator of transcription 5A | **1.72** | **1.65** |
| C3 | Complement component 3 | **1.53** | 1.36 |
| Il12rb1 | Interleukin 12 Receptor Subunit Beta | 1.51 | **3.63** |
| Fcgr3 | Fc receptor, IgG, low affinity III | -1.12 | **-1.53** |
| Fcgr4 | Fc receptor, IgG, low affinity IV | 1.17 | **-1.54** |
| Fcgrt | Fc fragment of IgG receptor and transporter | -1.26 | **-2.82** |
| Ccr7 | C-C chemokine receptor type 7 | **2.31** | **1.53** |
| Ccr2 | C-C chemokine receptor 2 | -1.19 | **-2.81** |
| Ccrl2 | C-C chemokine receptor-like 2 | **2.37** | **2.92** |
| Ifnlr1 | Interferon lambda receptor 1 | **2.14** | **1.49** |
| Catabolic Process |  |  |  |
| Autophagy |  |  |  |
| Hif1a | Hypoxia-inducible factor 1α | 1.13 | **1.73** |
| Ddit4 | DNA-damage-inducible transcript 4 | 1.13 | **2.08** |
| Bnip3 | BCL2 Interacting Protein 3 | 1.22 | **1.42** |
| Deptor | DEP Domain Containing MTOR Interacting Protein | -1.04 | **-2.21** |
| Mapk8 | Mitogen-activated *protein* kinase 8 | 1.00 | **1.68** |
| Traf6 | TNF receptor associated factor 6 | 1.20 | **1.44** |
| Cflar | CASP8 and FADD-like apoptosis regulator | **1.57** | **2.15** |
| Mras | Muscle and microspikes RAS | -1.18 | **-1.62** |
| Stx17 | Syntaxin 17 | -1.03 | **-1.48** |
| Atg14 | Autophagy Related 14 | 1.14 | **-1.48** |
| C9orf72 | C9orf72, member of C9orf72-SMCR8 complex | 1.14 | **1.46** |
| Map2k1 | Mitogen-activated protein kinase kinase 1 | 1.16 | **1.48** |
| Pik3cb | Phosphatidylinositol-4,5-bisphosphate 3-kinase catalytic subunit beta | 1.01 | **1.51** |
| Pik3r2 | Phosphoinositide-3-kinase regulatory subunit 2 | -1.15 | **-1.58** |
| Sqstm1 | Sequestosome 1 | **1.90** | **1.90** |
| Apoptosis |  |  |  |
| CD274 (PD-L1) | CD274 antigen | **1.71** | **2.03** |
| Faz | Fas Cell Surface Death Receptor | **1.52** | **1.63** |
| Tnfsf10 | Tumor necrosis factor superfamily, member 10 | - | **3.25** |
| Casp2 | Caspase 2, Apoptosis-Related Cysteine Peptidase | -1.18 | **-1.63** |
| Casp4 | Caspase 4, Apoptosis-Related Cysteine Peptidase | **1.83** | **2.27** |
| Casp6 | Caspase 6, Apoptosis-Related Cysteine Peptidase | -1.02 | **-1.43** |
| Casp7 | Caspase 7, Apoptosis-Related Cysteine Peptidase | 1.09 | **1.82** |
| Tnf | Tumor necrosis factor alpha | **2.41** | **4.75** |
| DAXX | Death Domain Associated Protein | **1.59** | **1.85** |
| Cflar | CASP8 and FADD-like apoptosis regulator | **1.57** | **2.15** |
| Birc3 | Baculoviral IAP repeat-containing 3 | 1,34 | **1.51** |
| Atm | Ataxia telangiectasia mutated | -1.21 | **-1.53** |
| Bcl2a1b | B cell leukemia/lymphoma 2 related protein A1b | 1.20 | **2.58** |
| Ctsf | Cathepsin F | -1.13 | **-1.83** |
| Ctsh | Cathepsin H | -1.09 | **-1.44** |
| Gadd45a | Growth arrest and DNA-damage-inducible 45 alpha | 1.42 | **1.83** |
| Dffa | DNA fragmentation factor, alpha subunit | 1.01 | **-1.59** |
| Endog | Endonuclease G | -1.10 | **-1.48** |
| Bbc3 | BCL2 binding component 3 | -1.05 | **-1.45** |
| Gadd45b | Growth arrest and DNA-damage-inducible 45 beta | **2.32** | **2.40** |
| Nfkb1 | Nuclear factor of kappa light polypeptide gene enhancer in B cells 1, p105 | 1.36 | **1.74** |
| Nfkbia | Nuclear factor of kappa light polypeptide gene enhancer in B cells inhibitor, alpha | **1.57** | **1.76** |
| Pik3r2 | Phosphoinositide-3-kinase regulatory subunit 2 | -1.15 | **-1.58** |
| Traf1 | TNF receptor-associated factor 1 | **2.03** | **2.17** |
| Map2k1 | Mitogen-activated protein kinase kinase 1 | 1.16 | **1.48** |
| Mapk8 | Mitogen-activated protein kinase 8 | 1.00 | **1.68** |
| Pik3cb | Phosphatidylinositol-4,5-bisphosphate 3-kinase catalytic subunit beta | 1.00 | **1.51** |
| Lysosome |  |  |  |
| Atp6v0d2 | ATPase, H+ Transporting, Lysosomal 38kDa, V0 Subunit D2 | -1.05 | **-1.70** |
| Ap1s2 | Adaptor Related Protein Complex 1 Subunit Sigma 2 | -1.21 | **-1.74** |
| Ap4m1 | Adaptor Related Protein Complex 4 Subunit Mu 1 | -1.01 | **-1.54** |
| *Lysosomal acid hydrolases* |  |  |  |
| *Proteases* |  |  |  |
| Ctsh | Cathepsin H | -1.09 | **-1.44** |
| Ctsf | Cathepsin F | -1.13 | **-1.83** |
| Lgmn | Protease, Cysteine, 1 (Legumain) | -1.06 | **-1.68** |
| *Glycosidases* |  |  |  |
| Naga | Alpha-N-Acetylgalactosaminidase | -1.05 | **-1.42** |
| Manba | Mannosidase, Beta A, Lysosomal | -1.10 | **-1.56** |
| Man2b1 | Mannosidase Alpha Class 2B Member 1 | -1.06 | **-1.49** |
| *Sulfatases* |  |  |  |
| Arsb | Arylsulfatase B | -1.25 | **-1.58** |
| Arsg | Arylsulfatase G | -1.00 | **-1.78** |
| Sgsh | N-Sulfoglucosamine Sulfohydrolase | -1.24 | **-2.45** |
| *Lipases* |  |  |  |
| Pla2g15 | Lysophospholipase 3 (Lysosomal Phospholipase A2) | -1.06 | **-1.73** |
| *Phosphatase* |  |  |  |
| Acp5 | Acid Phosphatase 5, Tartrate Resistant | 1.11 | **-1.41** |
| *Sphigomyelinase* |  |  |  |
| Smpd1 | Sphingomyelin Phosphodiesterase 1, Acid Lysosomal | -1.27 | **-1.51** |
| *Minor lysosomal membrane proteins* |  |  |  |
| Slc17a5 | Solute Carrier Family 17 (Anion/Sugar Transporter), Member 5 | 1.00 | **-1.77** |
| Slc11a1 | Solute Carrier Family 11 (Proton-Coupled Divalent Metal Ion Transporter), Member 1 | 1.29 | **1.64** |
| Laptm4b | Lysosome-Associated Transmembrane Protein 4-Beta | 1.26 | **1.66** |
| Abca2 | ATP-Binding Cassette, Sub-Family A (ABC1), Member 2 | -1.16 | **-1.66** |
| Sort1 | Sortilin 1 (Neurotensin Receptor 3) | -1.10 | **-1.49** |
| Antigen Processing and Presentation |  |  |  |
| MHC I Pathway |  |  |  |
| Tnf | Tumor necrosis factor alpha | **2.41** | **4.75** |
| Psme1 | Proteasome (Prosome, Macropain) Activator Subunit 1 (PA28 alpha) | 1.13 | **1.47** |
| Psme2 | Proteasome (Prosome, Macropain) Activator Subunit 2 (PA28 beta) | 1.29 | **1.87** |
| Hspa1a | Heat Shock Protein Family A (Hsp70) Member 1A | 1.79 | **1.73** |
| Hspa1b | Heat Shock Protein Family A (Hsp70) Member 1B | 1.66 | **2.22** |
| Hsp90aa1 | Heat Shock Protein 90 Alpha Family Class A Member 1 | 1.08 | **1.44** |
| Tap1 | Transporter 1, ATP Binding Cassette Subfamily B Member | 1.53 | **2.05** |
| Tap2 | Transporter 2, ATP Binding Cassette Subfamily B Member | 1.13 | **1.48** |
| Tapbp | Transporter associated with antigen processing (TAP) Binding Protein | 1.25 | **1.49** |
| Hspa5 | Heat Shock Protein Family A (Hsp70) Member 5 | 1.10 | **1.44** |
| MHC II Pathway |  |  |  |
| Lgmn | Legumain | -1.06 | **-1.68** |
| MHC complex |  |  |  |
| H2-K2 | Major histocompatibility complex K2 (MHC-Ia) | **1.98** | **1.92** |
| H2-M2 | Major histocompatibility complex M2 (MHC-Ib) | 1.21 | **2.29** |
| H2-Q1 | Major histocompatibility complex Q1 (MHC-Ib) | - | **2.56** |
| H2-Q2 | Major histocompatibility complex Q2 (MHC-Ib) | 1.43 | **1.69** |
| H2-Q5 | Major histocompatibility complex Q5 (MHC-Ib) | **1.92** | **2.10** |
| H2-Q6 | Major histocompatibility complex Q6 (MHC-Ib) | **1.87** | **1.65** |
| H2-Q7 | Major histocompatibility complex Q7 (MHC-Ib) | **1.95** | **1.65** |
| H2-T10 | Major histocompatibility complex T10 (MHC-Ib) | **1.48** | **1.62** |
| H2-T22 | Major histocompatibility complex T22 (MHC-Ib) | 1.16 | **1.59** |
| H2-T24 | Major histocompatibility complex T24 (MHC-Ib) | **2.70** | **1.84** |
| H2-Eb2 | Major histocompatibility complex E beta 2 (MHC-IIa) | - | **2.02** |
| H2-Oa | Major histocompatibility complex O alpha (MHC-IIb) | 1.00 | **1.53** |
| H2-DMa | Major histocompatibility complex DM alpha (MHC-IIb) | -1.09 | **-1.54** |

- Genes in bold lettering had their transcript levels significantly modulated (FC ≥ ± 1.4 and adjusted *p*-value < 0.05 as described in Materials and Methods). Positive and negative values represent genes with expression induced and repressed, respectively.
